# Alkali Hydrolysis of Sulfated Cellulose Nanocrystals: Optimization of Reaction Conditions and Tailored Surface Charge

**DOI:** 10.3390/nano9091232

**Published:** 2019-08-30

**Authors:** Jacobs H. Jordan, Michael W. Easson, Brian D. Condon

**Affiliations:** The Southern Regional Research Center, Agricultural Research Service, USDA, 1100 Robert E. Lee Blvd., New Orleans, LA 70124, USA

**Keywords:** agroindustrial waste, cellulose nanocrystals (CNCs), cotton, cellulose, nanocellulose, bioproducts, design of experiments

## Abstract

Cellulose nanocrystals (CNCs) are a biorenewable resource, which may be chemically modified to impart specific properties. Modified CNCs have found use in imaging applications, as rheology modifiers, polymer reinforcements, barrier and/or optical films, and nanocomposites. Nanoparticle dimensions of CNCs are typically 5–10 nm in width, with lengths of <100–300 nm. However, the physical properties are dependent upon the number and nature of the surface charge groups imparted during preparation. In the case of CNCs produced from sulfuric acid hydrolysis, the sulfated surface groups may be partially removed prior to further functionalization. This gives more available hydroxyls yet renders the CNCs less colloidally stable. Furthermore, conditions vary significantly and there is no consensus about the optimal conditions for partial removal of sulfate functionality or conditions developed to give specific surface charge. In the following, alkali hydrolysis of sulfate half-esters was quantified by conductometric titration of the strong acid groups, and using a design of experiments (DOE), optimal conditions were determined to produce CNCs with tailored surface charge.

## 1. Introduction

Cellulose nanocrystals (CNCs) are a biorenewable resource that is environmentally friendly, and possess unique physical properties such as high crystallinity and aspect ratio, large surface area, and a propensity to self-assemble into chiral nematic phases [1,2,3]. Thus, they have garnered such uses as reinforcing agents and additives in nanocomposites, polymers, gels, and emulsions [4,5,6,7,8,9,10,11,12]. Recently, cotton gin motes were shown to be an additional effective biomass source material for the extraction of cellulose and the production of CNCs [13]. Nanoparticle dimensions of CNCs are typically 5–10 nm in width with lengths of <100–300 nm [14]. There are a number of methods for producing CNCs. Hydrolysis with mineral acids, especially sulfuric acid (H_2_SO_4_), is by far the most common, although phosphoric acid (H_3_PO_4_) and hydrochloric acid (HCl) are also routinely used [15,16,17,18,19,20]. Hydrolysis with H_2_SO_4_ grafts anionic sulfate half esters (–OSO_3_^–^) onto the surface of CNCs, imparting them with a highly negative zeta (ζ) potential and colloidal stability in aqueous environments.

Extensive work has gone into investigating the hydrolysis conditions required to optimize yield, surface charge and nanocrystallite size [21,22,23]. For instance, thermally stable CNCs were produced by H_3_PO_4_ hydrolysis of Whatman filter paper [17]. The reaction conditions were subsequently optimized by Vanderfleet et al. using a design of experiments (DOE) to maximize the yield and surface functionalization of H_3_PO_4_ hydrolyzed CNCs [15]. There is considerably more variation in the reaction conditions and hence composition and morphology of CNCs produced from H_2_SO_4_ hydrolysis. Typical conditions found in the literature are 64 wt% H_2_SO_4_ at 45 °C for 30–45 min [4]. In many instances, the product yield, ζ-potential, and degree of surface functionalization—usually expressed as percent sulfur (%S), or mmol sulfate per kg of CNC (mmol·kg^−1^)—are often not reported. For instance, Lin and Dufresne used 65 wt% H_2_SO_4_ (45 °C, 60 min) to obtain CNCs with available surface –OSO_3_^–^ of 209 mmol·kg^−1^ and a ζ-potential of –40.7 mV; however, the yield was not reported [24]. Wang et al. developed a kinetic model (for bleached Kraft eucalyptus pulp), which indicated optimal conditions of 58 wt% H_2_SO_4_, 55 °C, 60 min based on constraints of maximal CNC yield at a given acid concentration and the minimal retention of cellulosic solid residues (CSR) and other decomposition products (vide infra). Experimental results were in agreement with the kinetic model and gave yields as high as 70%. At a lower acid concentration, insufficient depolymerization occurred and at higher concentrations, excessive degradation to monosaccharaides occurred. However, minimal product characterization was done, and only CNC and CSR yields were reported [22]. This agrees with the data provided by Chen et al. that correlated increased sulfation with increased temperature and acid concentration up to 62 wt% [23]. At low acid and temperature, sulfation occurs slowly (56 wt%, <50 °C). Cellulose sulfation occurred rapidly at temperatures >50 °C and 56–65 wt% H_2_SO_4_; sulfur contents were observed between 93–311 mmol·kg^−1^. However, at >62 wt% H_2_SO_4_, CNCs were further degraded into mono and oligosaccharides; thus, further sulfation reduced yields to <20% indicating typical conditions are not optimal [23]. Optimal conditions (55–58 °C, 120–250 min) gave yields of ~70% and resulted in sulfur contents of 210–250 mmol·kg^−1^.

The decomposition (hydrolysis) products of cellulose were investigated in sub-critical and super-critical water; the main degradation products produced are furfural and 5-hydroxymethyl-2-furfural (HMF) derivatives [24,25]. This is the cause of the brown color formation when CNCs are hydrolyzed under harsh conditions or for extended times. The reactions that lead to the formation of these degradation products have been discussed in detail previously [22,25]. Thus, to impart additional surface charge, nanocelluloses are post-functionalized by chemical treatment using chlorosulfonic acid to increase the sulfur content rather than increasing reaction time or acid concentration during preparation [24,26].

The inclusion of –OSO_3_^–^ groups has a marked effect on (i.e., decreases) the thermal stability of CNCs and limits their use in some applications (e.g., melt extrusion) when the –OSO_3_^–^ concentration is very high [4]. Moreover, –OSO_3_^–^ functional groups can impede the incorporation of other functionalities onto the CNC surface; for instance, the ability to graft photocatalysts onto CNCs was inversely correlated with the presence of sulfur on the CNC surface [27]. Abitbol et al. reported a correlation between the surface charge of CNCs and the concentration at which CNCs self-assembled into chiral nematic phases, including their viscosity and gelation properties [28]. The effective volume of CNCs (of identical dimensions) was found to be inversely proportional to the Debye length. As such, the volume increased with decreasing surface charge. Below a threshold charge of 0.27% S, axial end-to-end assemblies predominated.

It is, therefore, much more common to decrease the amount of surface –OSO_3_^–^ prior to post-functionalization. There are three potential pathways for the cleavage of sulfate half-esters on the CNC surface, one of which is favored dependent upon on the acidity of the conjugate acid (or nucleophile) used [29,30]. To accomplish this, there are several methods available: hydrothermal treatment, solvolytic cleavage, and acid-catalyzed or alkali desulfation treatments [31,32,33]. Lewis et al. demonstrated that a hydrothermal treatment of dilute suspensions of sulfated CNCs above 80 °C caused surface desulfation and resulted in the formation of CNC hydrogels [33], while Lin et al. employed an alkali treatment prior to the incorporation of surface cationic groups to prepare alginate based CNC hydrogels [34]. Dorris and Gray showed that in a mixture of glycerol/water, CNC suspensions were susceptible to auto-catalyzed desulfation as a requisite for gel formation; >30% desulfation occurred in 8 h at 70 °C [35].

Jiang et al. observed that the acid-catalyzed desulfation of CNCs resulted in increased degradation products and the incomplete removal of surface –OSO_3_^–^ groups, while solvolytic desulfation gives the CNC product as a pyridinium salt [32]. The former was not quantified while the latter does not allow for tailored surface charge. Acid-catalyzed desulfation was employed to achieve ~60% desulfation of H_2_SO_4_-hydrolyzed CNCs; the partially desulfated CNCs were functionalized with a fluorescent marker at ~2.5 times the concentration of sulfate CNCs [36]. Acid-catalyzed desulfation was shown to proceed rapidly and stabilize after 1–2 h in 2.5 N HCl for 1–10 h at ~98 °C [37]. Ellebracht et al. used an acid-catalyzed desulfation as the first linear step in a multi-step desulfation-functionalization approach, to generate amine coupled CNCs as acid-base catalysts for aldol condensations [38]. Pandey et al. used both acidic and alkali desulfation procedures to investigate the influence of surface charge and aggregation state on the stability, microstructure and rheology of Pickering emulsions [39].

Further use of alkali hydrolysis of –OSO_3_^–^ groups by Zoppe et al. allowed the post-functionalization of CNCs to give biomimetic nanostructures [40]. In this case, the protocol (1 wt% dispersions, 1 M NaOH, 5 h) was selected to minimize mercerization and changes in crystallinity. The same method was employed to impart organic spacers between the CNC surface and functional groups for bioactive nanocomposites [41]. An alkali treatment was employed to reduce the surface charge (and hence –OSO_3_^–^ groups) of CNCs, to control the nucleation and growth of silver nanoparticles (AgNP) [42]. The size-distribution was found to be dependent upon the number of –OSO_3_^–^ groups and optimal within a specific range; too much –OSO_3_^–^ gave a large size distribution while too little led to larger AgNP. Table 1 summarizes many of the reported conditions for acid-catalyzed and alkali desulfation of CNCs.

As Table 1 indicates, conditions range from the very mild to the very harsh; there is considerably less variation in the conditions employed for acid-catalyzed desulfation, in part because it has been utilized for nearly two decades, and is the more thoroughly studied/understood. However, acid-catalyzed desulfation has the undesirable drawback of potential changes in the physical properties (e.g., dimensions and crystallinity) of the CNCs, due to the shortening of the cellulose chains from additional glycosidic bond cleavage. Moreover, acid-catalyzed desulfation also has a greater energy requirement than alkali hydrolysis (e.g., 80–100 °C vs. 50–65 °C) and results in CNCs in their acid form which are susceptible to further (unintended) degradation during storage [46].

Although numerous methods are used to prepare CNCs, successful surface modification requires CNCs of moderate thermal and colloidal stability (i.e., sufficient surface charge) but with minimal surface functionalization, that is, minimal amounts of –OSO_3_^–^ when prepared from H_2_SO_4_ hydrolysis [49]. Thus, it is the goal of this work to establish optimal conditions to target desired functionality on the CNC surface (e.g., < 100 mmol·kg^−1^) given a known starting value of sulfate (e.g., 200–400 mmol·kg^−1^).

## 2. Materials and Methods

### 2.1. Raw Materials

Cotton gin motes were obtained from the USDA Research Facility in Stoneville, Mississippi. Chemicals and supplies were purchased from MilliporeSigma Corporation, or VWR-USA and were used as received. All water sources used deionized water with a maximal conductivity of ≤1.0 µS·cm^−1^, typically ~0.30–0.50 µS·cm^−1^. Ice water was generated from a house filtration system with a conductivity of 25–30 µS·cm^−1^.

### 2.2. Nanocrystal Preparation

Cellulose nanocrystals were prepared as previously described [13]. Briefly, cotton gin motes were mechanically ground to 40 mesh with a knife mill (Wiley Mill E3300, Eberbach Corporation, Belleville, MI, USA). Celluloses were extracted by subsequent alkali and bleaching treatments. For the alkali treatment, a 4% solution of sodium hydroxide (NaOH) was used for 2 h at 70 °C. For the bleaching treatment, an acidified sodium chlorite (NaClO_2_) solution (1.0% acetic acid (*v*/*v*) and 0.25% NaClO_2_ (*w*/*v*)) was used for 2 h at 75 °C; bleaching was repeated until the fibers were fully white. In both instances, the fiber to liquor ratio was 1:40 (*w*/*v*). The recovered fibers were washed thoroughly with water until the eluent was near neutral (pH ≈ 6–7) and then oven-dried (70 °C) to a constant mass.

Hydrolysis of cellulose extracted from gin motes was conducted for 30–150 min at 45–60 °C with 55–65 wt% H_2_SO_4_ at a material to liquid ratio of 1:15 (*w*/*v*). The resulting suspension was quenched four-fold by dilution with ice water and then washed until pH ≥5 by successive centrifugation cycles at 16,000 *g* for 15 min each cycle. The crystals were then dispersed using a 750 W ultrasonic processor (Vibra-Cell probe sonicator, VCX-750, Sonics & Materials, Newtown, CT, USA) with a power amplitude of 60% (20 kHz frequency) for 5 min. The obtained suspension was centrifuged at 5000 rpm for 5 min (to remove larger particulates and contaminates), filtered under vacuum with a Whatman glass microfiber filter (grade GF/F, 0.7 μm) and dialyzed using a regenerated cellulose dialysis tubing (MW cutoff 10,000) for several days until the solution conductivity stabilized for two successive bath changes (measured value <2 µS·cm^−1^). The suspension was subsequently stored in a sealed container and refrigerated (4–8 °C) between uses. The yield and concentration of the nanocrystals was determined gravimetrically.

### 2.3. Surface Desulfation of H_2_SO_4_-Hydrolyzed CNCs

An alkaline (NaOH) treatment was employed to obtain the surface desulfation of H_2_SO_4_-hydrolyzed CNCs. The maximum final concentration of NaOH used in this work was kept ≤8 wt% (≈2 M). For the alkali treatment, concentrated stock CNC suspensions (~3.2 wt%) were diluted with an appropriate volume of water before the addition of a variable amount of concentrated NaOH solution (7.30 M). The final concentration of obtained CNC suspensions was 0.5–2.0 wt%, and NaOH was 0.0–2.0 M. Solutions were heated to 25–75 °C for 0.0–6.0 h. At the conclusion of the reaction, suspensions were quenched by the addition of ice water to bring the volume to 49 mL and 1 mL of 4.0 M sodium chloride (NaCl) was added to aid flocculation. Samples were centrifuged (16,000× *g*, 15 min), the supernatant decanted and washing repeated. After, the CNC pellets were suspended by probe sonication with 30 s pulses and exhaustively dialyzed until the solution conductivity stabilized (typically, ~1.0 µS·cm^−1^).

### 2.4. Conductometric Titration

The sulfate half-ester content was measured by conductometric titrations as described previously, with minor modifications [50,51,52]. The conductometric titrations were performed on CNCs in acid form (–OSO_3_H). CNCs were converted from their sodium (Na^+^) to their acid from by chromatography over a large excess of Dowex^®^ Marathon™ C Hydrogen from ion-exchange resin (23–27 mesh). While the concentration of the stock CNC suspensions varied, the concentration must be known accurately for meaningful determinations of the surface charged groups. For the stock CNC suspension, 5 mL of ~1.0 wt% CNCs was diluted to 99 mL and to this was added 1 mL of 100 mM NaCl. For analysis of hydrolysis experiments, the obtained CNC suspensions were diluted to 0.02 wt% suspension in 100 mL of water containing 50 µM NaCl as added electrolyte. Throughout each titration, the conductivity was continuously monitored, and 100 µL aliquots of standardized 1.03 mM NaOH were added over 45–60 min. The volume-corrected conductivity was plotted using OriginPro 2018b (OriginLab, Northampton, MA, USA) and the equivalence point was determined by the intersection of least-squares regressions from the positive and negative sloped regions. Data was collected in (at least) triplicate for each sample.

### 2.5. Atomic Force Microscopy

The atomic force microscopy (AFM) measurements were performed with an Agilent 500 atomic force microscope for native CNCs only. Data was collected in contact mode using a triangular-shaped Pyrex nitride cantilever with a gold reflex coating and silicon nitride tips (0.32 N/m force constant, 67 kHz resonance frequency, NanoWorld, Neuchâtel, Switzerland). For the determination of the CNC length, and diameter (height), mica discs (V1 AFM Mica Discs, 20 mm, TedPella, Inc., Redding, CA, USA) were pretreated with 100 µL of a poly-L-lysine solution (0.01 wt%) and rinsed thoroughly with water after two minutes and blown dry with a stream of Argon. CNC suspensions (0.01 wt%) were applied by the drop cast method and rinsed after two minutes and blown dry. AFM height measurements were determined by use of the section analysis tool provided with the AFM software (Agilent Picoview 1.14, Chandler, AZ, USA) on a 4 × 4 µm image from at least 100 individual observations. Image J (National Institutes of Health, Bethesda, MD, USA) was used to determine the CNC length. Results were then fit with a Gaussian function using OriginPro2018b to determine the mean length and height of the CNCs.

### 2.6. ζ-Potential

The CNC suspensions were diluted to 0.20 wt% and filtered through a 0.45 µm polyvinylidene fluoride (PVDF) filter to remove any larger particulates and dust [19]. The suspensions were analyzed for electrophoretic mobility and hence the ζ-potential using a Malvern Zetasizer Nano (Malvern Panalytical Ltd., Malvern, United Kingdom). ζ-potential measurements were collected with a 5 mM NaCl buffer; the addition of some salt is necessary to give an accurate ζ-potential measurement. The ζ-potential of CNC samples was measured using the Smoluchowski approximation of Henry’s function for aqueous dispersions with a suitable electrolyte concentration, such that the electric double-layer thickness around the CNCs is thin compared to the particle size. CNC dispersions were measured at 25 °C with each measurement consisting of at least 15 cycles. Data was collected in triplicate and the error is presented as the standard deviation from individual measurements.

### 2.7. Design of Experiments (DOE)

In a series of thirty-two experiments, time (*t*), temperature (*T*), NaOH concentration (molarity) and CNC wt% (factors) were simultaneously varied within a prescribed range and the resulting CNC absolute sulfate concentration (response) was measured. A software program by Stat-Ease, Inc., Design Expert (Version 9.0.6.2, Minneapolis, MN, USA), provided statistical analysis of the experimental response, based upon the four input process factors. A quadratic model was selected from the software program and a regression analysis performed to eliminate insignificant terms, which strengthen the model. The experimental data passed standardized diagnostic tests, including the normal plot of residuals and Box–Cox (See: Supplemental Information, Figures S5–S8).

## 3. Results and Discussion

The cotton gin motes used have 67.4% cellulose content [13]. Hydrolysis of the extracted cellulose with H_2_SO_4_ gave yields of 14–43% (Table 2). Nanocrystal dimensions were 4–10 nm in width and 100–200 nm in length (See Supporting Information, Figures S1‒S3, for details). Generally, shorter reaction times, or a lower acid concentration, resulted in slightly larger crystallite sizes, while longer reaction times or a high acid concentration produced smaller crystals with a higher concentration of –OSO_3_^–^ [22].

**Figure 1:**
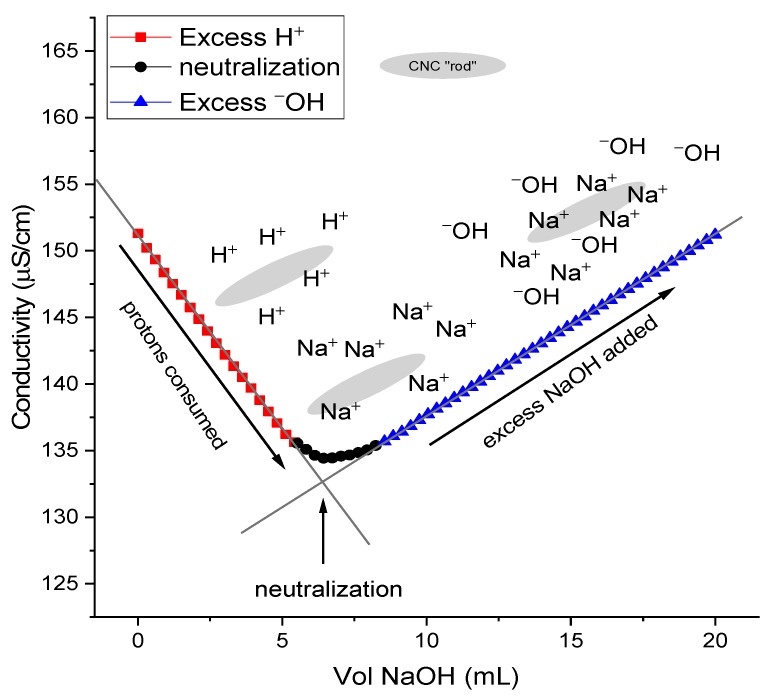
Representative example of conductometric titration from CNC batch 3 (~0.032 wt%) against 1.03 mM NaOH in 1 mM NaCl.

The sulfate half-ester content (–OSO_3_^–^) was 134–308 mmol·kg^−1^ as measured by conductometric titration (Figure 1). This falls well within the typical values for H_2_SO_4_-hydrolyzed CNCs of 80–350 mmol·kg^−1^ [53]. For conductivity measurements, a strong acid–ion exchange column was used following dialysis; this is to ensure the full protonation of CNCs. If dialysis is used alone, or after treatment with ion-exchange resins, low sulfate concentrations are detected [50,51]. For instance, in two examples using acid hydrolysis, the initial surface sulfate ~0.14–0.16 e/nm^2^ (corresponding sulfate is 40–44 mmol·kg^−1^) is unusually lower than typical values (80–350 mmol·kg^−1^). Moreover, after acid-catalyzed desulfation, a complete absence of sulfate was indicated by a lack of negative slope in the conductometric titrations; in both instances, dialysis was conducted prior to treatment with a mixed-bed resin, while no strong acid resin was employed, undoubtedly, as a direct consequence of using an acid-catalyzed desulfation. Thus, the obtained results are erroneously lower than expected [43,54].

Beck et al. showed that CNCs in acid form are less stable due to auto-degradation products of the sulfate half-esters; heating a concentrated suspension at elevated temperatures resulted in significant desulfation (~50%) in as little as 2 h [46]. Even mild heating (40–50 °C) was shown to induce an acid-catalyzed desulfation over several days for aqueous suspensions of CNCs in protonated form [3]. Thus, it is critical to convert CNCs to their sodium form prior to heating and to ensure any residual acids are removed, as adventitious protons derived from the sulfate half-esters can lead to auto-catalyzed acidic degradation of the –OSO_3_^–^ groups, while a slight excess of cations can lead to erroneous measures of initial –OSO_3_^–^ concentration after ion exchange [50,51]. Exhaustive dialysis measured by a conductivity probe is necessary, rather than relying on the solution pH. Even small contaminates of H_3_O^+^ can significantly alter the solution conductivity (the molar conductivity of the hydronium ion is ~350 S·cm^2^·mol^−1^). Significant differences are observed after a few µmol added H_3_O^+^ (See Supporting Information, Figure S4).

By contrast, CNCs in sodium form are stable; Beck et al. showed no desulfation over several days in the absence of excess ^–^OH, and a statistically insignificant amount (within error) when dilute NaOH was employed in a few hours [46]. This is in contrast to the report by Lokanathan, where Δ(OSO_3_^–^) ≈ 15% when using as little as 10 mM NaOH over 30 min [42]. The stability of CNCs was tested, and experimental results (#1–2) corroborate the results obtained by Beck et al. and indicate 50–60% desulfation of CNCs in acid form at 75 °C, no significant desulfation at room temperature, and no significant changes in –OSO_3_^–^ from work-up for CNCs in sodium form (exp #3) or after elevated heating (exp #4). The results are shown in Table 3.

To remove sulfate half-esters, an alkali (NaOH) treatment is used [24,31,45]; the yields from alkali treatments are typically 60–80%. A single round of alkali treatment is insufficient to fully remove sulfate half-esters from CNCs [45], however, by varying the concentration of the alkali treatment, or the treatment time, CNCs with a gradient sulfate substitution were obtained [24]. In order to investigate the desulfation process, the present research employed a DOE approach wherein optimal desulfation occurred based upon time, temperature, NaOH concentration and CNC wt% as input variables (factors). The resulting desulfation (response) provided a means to model the optimal experimental conditions (vide infra). Notably, under the conditions used by Lin and Dufresne, no changes to the physical properties or crystallinity of the isolated products was observed [24]. Thus, detailed physical, and chemical analysis of the products isolated from this DOE was not performed. Stock CNCs were analyzed for available –OSO_3_^–^, particle size by AFM, and ζ-potential, while products were analyzed for –OSO_3_^–^ and ζ-potential as response variables.

Table 1 was used to select conditions for desulfation reactions [24,31,45]. Five experiments were conducted to vary the reaction time, temperature, and NaOH concentration: 30–60 °C, 2.5–15 h, 0.5–1.0 M NaOH. Table 4 shows the results of alkali desulfation under these conditions. Alkali treatment at elevated temperatures (2.5 h, 0.5–1.0 M NaOH, 60 °C) resulted in the removal of about one-third of surface sulfate groups, yet showed no dependence on the concentration of NaOH used (exp #1–2). By contrast, doubling the reaction time increased the efficiency of the reaction by ~50% (exp #3). Even under mild conditions (5–15 h, 1.0 M NaOH, 30 °C) approximately one-fifth of the available sulfate could be removed. There was no increase in sulfate removal beyond five hours, however.

Kloser and Gray showed that harsher reaction conditions (1.7 M NaOH, 85°C, 72 h) resulted in a yield of only 6% and removal of about 80% of the sulfur content [45]. By contrast, successive rounds of desulfation can achieve gradient sulfate content without significantly compromising yield (Table 5); on average one-third of the available sulfate half-esters were hydrolyzed during each round of desulfation. This has the unfortunate drawback of significantly increasing time, as the work-up and isolation of the CNCs between each cycle of desulfation is cumbersome. It is therefore a necessity to establish optimal conditions to facilitate the effective removal of sulfate half-esters to arrive at a preferred final concentration of –OSO_3_^–^ that minimizes the use of reagents, and ultimately simplifies or shortens work-up and energy requirements.

**Figure 2:**
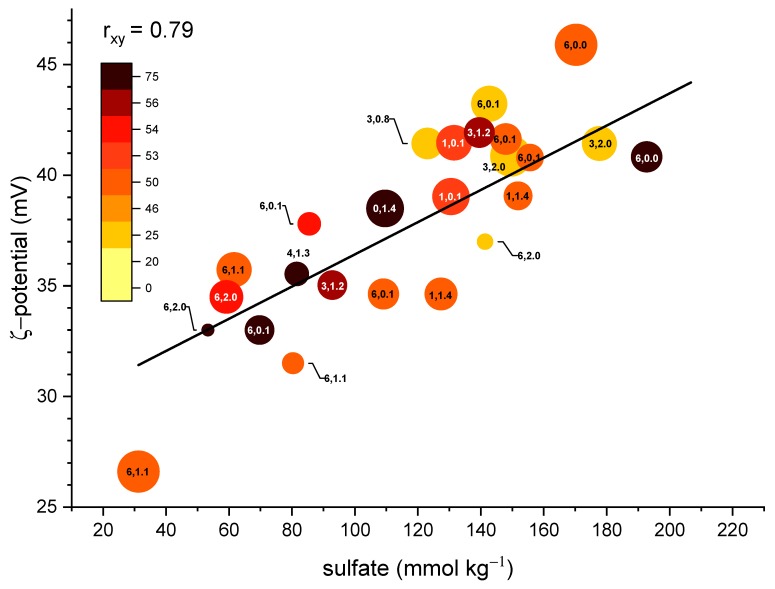
Plot of ζ-potential (mV) versus –OSO_3_^–^ functionalization (mmol·kg^−1^). The error in the measurement is indicated by the size of the spheres; the color bar represents the reaction temperature (°C) for the hydrolysis reaction, while the numbers represent the time in hours and the molar concentration of NaOH, (e.g., 3 h, 1.0 mol·L^−1^ is (3, 1.0)). *t* = 0 masked for clarity. Pearson’s correlation of the linear fit “r_xy_” = 0.79.

Based on these results and literature precedent, the bounds for the DOE were set at *T* ≤ 75 °C, and *t* ≤ 6 h, to allow some reactions to (presumably) reach equilibrium prior to work-up and also to minimize product losses due to glycosidic bond cleavage at elevated temperature or longer reaction times [45]. Additionally, the final CNC concentration was allowed to vary from 0.5 to 2.0 wt%, and the NaOH concentration was maintained at ≤8 wt% (2.0 M) to prevent possible mercerization of the cellulose chains. This corresponds to a maximum molar ratio of NaOH to –OSO_3_^–^ of about 2000:1 on the more dilute (0.5 wt%) samples. The maximum molar ratio of NaOH to –OSO_3_^–^ was limited to about 500:1 on the more concentrated samples to fall within the experimental bounds. It was not possible to perform the experiments on a larger diluted sample to increase the available NaOH and still test the requisite CNC concentration for the DOE. The results are given in Table S1 and summarized in Figure 2.

For conductometric titrations, the addition of some electrolyte improves the stability of the measurements for dilute solutions, and CNC suspensions are commonly measured in the presence of 1 mM NaCl as were the stock solutions (vide supra) [51]. However, higher concentrations of CNCs and electrolyte resulted in flocculation and irreproducible results in data collection for some experiments. Thus, the final concentration of the CNC suspensions for conductometry experiments obtained from the DOE was fixed at 0.02 wt% and the electrolyte concentration held constant at 50 µM for analysis of alkali-hydrolyzed CNCs, to prevent flocculation and maintain the same electrolyte concentration throughout the experiments.

Returning to Figure 2, there is not an apparent simple linear relationship between any two terms. However, there is a positive correlation between the concentration of –OSO_3_^–^ and the ζ-potential. Generally, there is a grouping of high remaining –OSO_3_^–^ at low temperature, and for reactions that occurred at similar temperature profiles, Δ(–OSO_3_^–^) correlates with longer reaction times and/or a higher NaOH concentration.

A quadratic model was used to fit the data; the DOE found that time, temperature and a two-factor interaction between time and NaOH concentration were significant. Additionally, the significance of the NaOH concentration was nonlinear; this could be explained in part by a visual observation of rapid flocculation of the CNCs at very high NaOH concentration, promoted by Na^+^ condensation on the negatively charged CNC surface. Thus, intermediate concentrations of NaOH are preferred (vide infra), and below this regime, insufficient NaOH concentration attenuated the observed response. Furthermore, an equation (Equation (1)) was generated which predicted the desulfation response (DS), based upon input responses and significant model terms (Equation (1), See Supporting Information, Tables S2–S3, Equation S1), where *S* is a constant which is dependent upon the initial sulfate concentration, *t* is the time in min, *T* is the temperature (°C), and *C* is the equivalence of NaOH:

*DS = S* − 5.68 × 10^−3^*t* − 3.26*T* − 2.08 × 10^−4^*tC* +6.73 × 10^−5^*C*^2^ (1)

**Figure 3:**
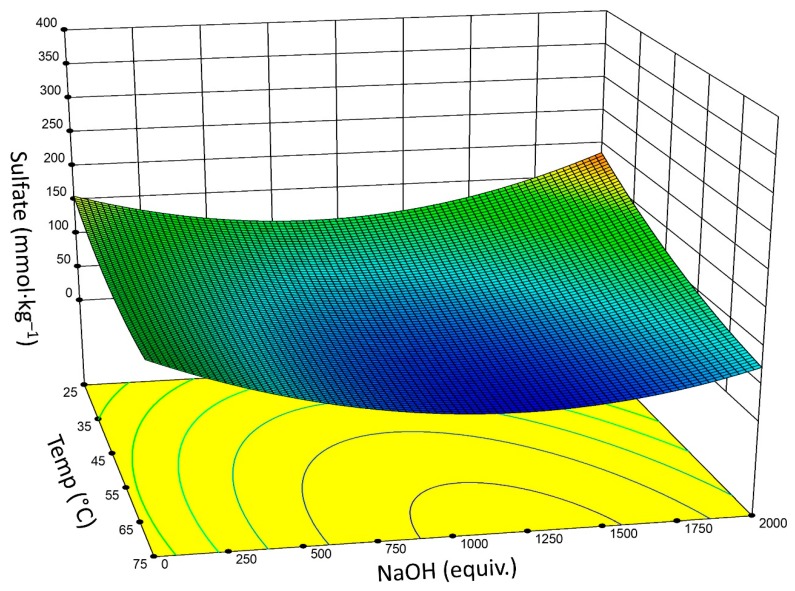
Plot of temperature (°C) versus NaOH concentration (shown as equivalents NaOH per µmol –OSO_3_^–^) with associated output –OSO_3_^–^ functionalization (mmol·kg^−1^). (*t* *=* 6 h).

Generally, within a given temperature range, increasing temperature and/or time results in an increase in –OSO_3_^–^ hydrolysis. Moreover, since the hydrolysis is an equilibrium between the sulfated and desulfated CNCs, higher working concentrations of CNCs (increased wt%) elevated temperatures, and increased NaOH concentration (to a point), favor desulfation. Although not considered a significant factor, CNC wt% and thus the initial concentration of –OSO_3_^–^ was used as a model term. At lower concentration of CNCs greater equivalents of NaOH are needed, while at higher concentrations, the optimal window is shifted towards lower equivalents NaOH (the blue region is shifted to the left in Figure 3). At a set reaction time (*t* = 6 h) there is a broad range of conditions to provide sufficient hydrolysis of sulfate half-esters (Figure 3); however, these conditions are not always optimal. For instance, given a 200 mL, 2.0 wt% suspension containing CNCs with ~200 mmol·kg^−1^ –OSO_3_^–^, maximal desulfation would be obtained at ~1000 equivalents NaOH per available –OSO_3_^–^ and *T* ≥ 60 °C, which corresponds to a 4 M (16 wt%) solution of NaOH. Much more manageable conditions can be employed: 0.8 wt%, 1.6 M NaOH, 60 °C, and 6 h. Thus, from ~0.75–2.0 wt%, the amount of NaOH can vary from 1500–500 equivalents per unit –OSO_3_^–^. Under these conditions there was little observed difference for reactions with *T* ≥ 60 °C, while lower temperatures attenuate the hydrolysis of the sulfate half-esters. Furthermore, there is a mild dependence on the initial concentration of CNCs, albeit more concentrated samples (greater wt%) undergo a greater absolute change in sulfate concentration upon hydrolysis. This explains the contradiction in results obtained by Beck et al., and Lokanathan et al. which showed 8% and 22% hydrolysis, respectively, with 100 mM NaOH [42,46]. In this instance, the former reactions were carried out under comparatively mild conditions (50 °C, 0.56 wt%, 160 min), while the latter were used more stringent conditions (65 °C, 1.0 wt%, 30 min) resulting in a greater observed hydrolysis despite the much shorter reaction time.

**Figure 4:**
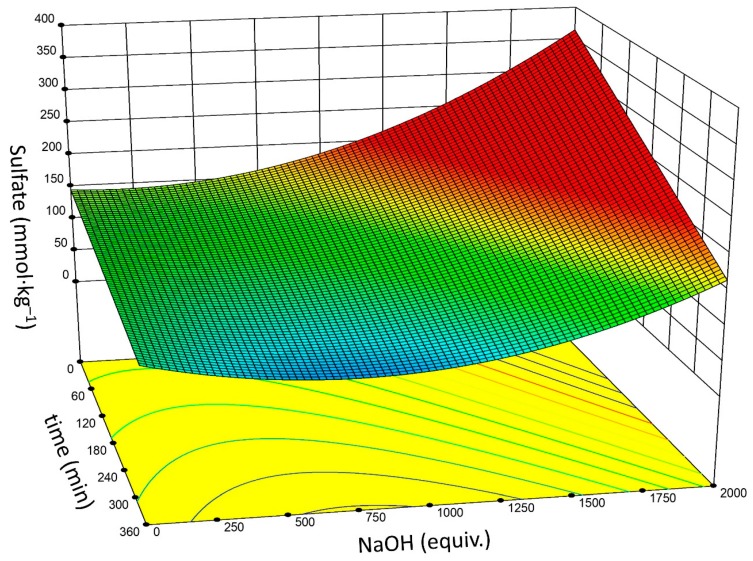
Plot of time (min) versus NaOH concentration (shown as equivalents NaOH per µmol –OSO_3_^–^) with associated output –OSO_3_^–^ functionalization (mmol·kg^−1^). *T* = 60 °C.

Most reported alkali desulfation reactions use 0.5–2.0 M NaOH. However, the DOE results confirm several examples where significantly reduced (≤0.1 M) NaOH concentrations were used [24,42]. Sufficient concentration of NaOH is a necessary, but insufficient requirement for hydrolysis of sulfate half-esters; temperature and time are weighted heavily. It is likely, given the results of the DOE, gradient sulfate degrees (vide supra) were primarily the result of changes in the reaction time (1–3 h), and not from changes to the concentration of the NaOH (0.5–2.0 M), which was always present in a large excess (~60–250 equivalents per mmol –OSO_3_^–^) [24]. At a fixed temperature of 60 °C, there is a narrower range of optimal conditions to minimize remaining –OSO_3_^–^ (Figure 4), and yet still covers a gamut of possible selections. Specifically, 250–1250 equivalents NaOH, and t ≥ 5 h provides for maximal desulfation.

However, it is apposite to note, while these conditions are optimal in terms of maximal Δ(–OSO_3_^–^), increased concentration of NaOH increases costs and time associated with work-up and purification, while increasing temperature results in significantly increased energy requirements. There is very little difference observed in desulfation above a threshold temperature of ~60 °C; additionally, while some amount of NaOH is necessary for hydrolysis, high concentration is not required, especially since complete desulfation is often not the goal (nor possible).

If parameters are selected at a fixed temperature (60 °C) and CNC concentration (2.0 wt%), and the DOE solved to minimize NaOH concentration, maximize Δ(–OSO_3_^–^), and time allowed to float freely (0–6 h), a number of possible solutions within 1–2% Δ(–OSO_3_^–^) were obtained. Under these restrictions, optimal conditions are 60–120 equivalents NaOH and 3–6 h to obtain 58–62% Δ(–OSO_3_^–^). Conditions of 0.13 M NaOH, 0.78 wt% CNC suspension (–OSO_3_^–^ = 211 mmol·kg^−1^) were selected, in this instance, corresponding to 80 equivalents of NaOH per unit –OSO_3_^–^, and a total solution volume of 50 mL was used. Gratifyingly, results from this test condition were Δ(–OSO_3_^–^) = 57%, which corresponds to –OSO_3_^–^ of 211 and 91.6 mmol·kg^−1^ initial and final values, respectively. This is in very close agreement to the expected results from the DOE.

## 4. Conclusions

1. Hydrolysis of sulfate half-esters on the CNC surface occurred over a broad range of different conditions: [NaOH] (<0.1 M to >2.0 M), CNC wt% (~0.5 to ≥2.0 wt%) and time (>0 to ≤6 h).
2. Above 0.1–0.2 M NaOH there is only a minor observed difference in overall efficacy of –OSO_3_^–^ removal.
3. Based upon DOE analysis, reaction time, temperature, and NaOH concentration are significant factors for effective sulfate half-ester removal. There is a two-factor interaction between reaction time and NaOH concentration. The significance of the NaOH concentration is non-linear.
4. Optimal conditions may vary depending on the initial and targeted sulfate concentration: 60–120 equivalents NaOH and a reaction time of 3–6 h gives Δ(–OSO_3_^–^) of ~60%.
5. The traditional conditions (1.5 M NaOH, 60 °C, 5 h) typically remove about one-third to one-half of the available –OSO_3_^–^ groups, dependent upon the selected concentration of CNCs (wt%).
6. More desirable conditions still yield colloidally stable CNCs at significantly lower use of NaOH, thus improving work-up.

## Figures and Tables

**Table 1:** Literature reported methods for acidic and alkaline cellulose nanocrystals (CNC) desulfation methods.

Method	Conditions (Final) ^1^	–OSO_3_^–^ (mmol·kg^−1^)		Reference
		Initial	Final	
Acidic	0.97 wt%, 0.024 M HCl, 80 °C, 2.5 h	293	191	[32] ^1^
	×2		153	
	×3		103	
	×4		58	
	×7		55	
	4.39 wt%, 0.025 M HCl, 80 °C, 20 h	148 ± 12	64 ± 8	[36]
	4.5 wt%, 0.025 M HCl, 80 °C, 20 h	125	47	[27] ^1^
	0.4 wt%, 2.5 N HCl,	44	n/a ^3^	[43] ^1^
	0.5 wt%, 0.05 M HCl, 80 °C, 24 h	280	120	[38]
	2.0 wt%, 2.5 M HCl, 100 °C, 5 h	~430	~50	[39] ^4,5^
Alkaline	9 wt%, 2.0 M NaOH, 65 °C, 5 h	130 ± 95	n/a ^3^	[31,44] ^2^
	2.78 wt%, 1.0 M NaOH, 60 °C, 5 h	240	80	[45]
	2.78 wt%, 1.7 M NaOH, 85 °C, 72 h	240	40	
	2 wt%, 0.1 M NaOH, 23 °C, 20 min	234	222	[46] ^5^
	0.56 wt%, 0.1 M NaOH, 50 °C, 20 min	244	240	
	0.56 wt%, 0.1 M NaOH, 50 °C, 160 min	244	225	
	0.55 wt%, 0.85 M NaOH, 50 °C, 20 min	240	228	
	0.56 wt%, 0.85 M NaOH, 50 °C, 180 min	240	229	
	1.33 wt%, 0.17 M NaOH, 60 °C, 1 h	209	166	[24] ^1^
	1.33 wt%, 0.33 M NaOH, 60 °C, 1.5 h	209	144	
	1.33 wt%, 0.50 M NaOH, 60 °C, 2 h	209	90.6	
	1.33 wt%, 0.67 M NaOH, 60 °C, 3 h	209	56.3	
	1.0 wt%, 1.0 M NaOH, 60 °C, 5 h	220	40	[40,41,47]^2^
	1.0 wt%, 0.01 M NaOH, 65 °C, 30 min	~194	~165	[42] ^4^
	1.0 wt%, 0.1 M NaOH, 65 °C, 30 min	~194	~152	
	1.0 wt%, 0.5 M NaOH, 65 °C, 30 min	~194	~142	
	5.0 wt%, 1.5 M NaOH, 65 °C, 5 h	~219	~125	[48] ^1,4^
	2.0 wt%, 2 M NaOH, 65 °C, 5 h	~430	~190	[39] ^5^
	1.45 wt%, 1.0 M NaOH, 60 °C, 2.5 h	150 ± 15	62 ± 1	[13]

^1^ Conditions reflect the final adjusted concentration of CNCs and reagent (e.g., NaOH or HCl); for instances when the protocols called for mixing two or more components at a given concentration, values are representative of the final reaction conditions employed. ^2^ The sulfate half-esters were not protonated prior to conductometric titration, so values are not absolute. ^3^ Quantification of the –OSO_3_^–^ surface groups was not performed or detected. ^4^ Values indicated with a tilde operator (~) are calculated from elemental sulfur (%S) or surface charge density σ (e/nm^2^). ^5^ Measured using inductively coupled plasma (ICP) techniques: ICP atomic emission spectroscopy (ICP-AES) or triple quadrupole ICP-MS (ICP-QQQ).

**Table 2:** Conditions, yield, and properties of H_2_SO_4_-hydrolyzed CNCs.

Batch (#)	H_2_SO_4_ (wt%)	Temp (°C)	Time (min)	–OSO_3_^–^ (mmol·kg^−1^)	ζ-Potential (mV)	Yield (%)
1	55	60	150	155	–31.5 ± 1.5	43
2	65	55	60	197	–40.8 ± 0.7	21
3	65	45	90	211	–41.1 ± 1.5	32
4	65	60	90	308	–45.3 ± 1.0	14
5	62	50	30	134	–41.5 ± 1.1	39

**Table 3:** Comparison of literature, and experimental, auto-catalyzed in situ desulfation reactions.

Sample	CNC (wt%)	Temp (°C)	Time (h)	–OSO_3_^–^ (mmol·kg^−1^)		Ref. or exp #
				Initial	Final	
H-CNC	3.8	70	120	265 ^2^	90 ^2^	Ref. [46]
H-CNC	2.8	85	72	275 ^1^	103 ^1^	
H-CNC	4.0	100	2	217 ^2^	108 ^2^	
Na-CNC	2.8	85	72	275 ^1^	275 ^1^	
Na-CNC	3.8	70	120	265 ^2^	254 ^2^	
H-CNC	0.50	25	6	211 ^1^	204 ^1^	#1
H-CNC	0.50	75	6	197 ^1^	87 ^1^	#2
Na-CNC	0.50	25	0	211 ^1^	204 ^1^	#3
Na-CNC	0.50	75	6	197 ^1^	193 ^1^	#4

^1^ Measured by conductometric titration. ^2^ Measured by ICP-AES.

**Table 4:** Preliminary desulfation reactions to set the bounds of the design of experiments (DOE).

Exp (#)	Time (min)	Temp (°C)	[NaOH] (M)	[CNC] (wt%)	Yield (%)	–OSO_3_^–^ (mmol·kg^−1^)		Δ(–OSO_3_^–^) (%)
						Initial	Final	
#5	150	60	0.5	1.44	88	155	109	30%
#6	150	60	1.0	1.44	83	155	105	32%
#7	300	60	1.0	1.44	87	155	86	45%
#8	300	30	1.0	1.44	83	155	121	21%
#9	900	30	1.0	1.44	90	155	122	22%

**Table 5:** Successive alkali desulfation of CNC suspension.

Sample	CNC (wt%)	–OSO_3_^–^ (mmol·kg^−1^)		Δ(–OSO_3_^–^)
		Initial	Final	(%)/net
#10	0.72	308	213	31|31
×2	0.58	213	162	24|47
×3	0.45	162	103	36|67
×4	0.25	103	69	33|78

In all instances the reaction conditions were: 1.5 M NaOH, 60 °C, and 6 h; the starting concentration of the CNC and the –OSO3– varied, reaction volume (50 mL) was kept consisten.

## Supplementary Materials

The following are available online at https://www.mdpi.com/2079-4991/9/9/1232/s1, Figure S1 to Figure S3: Characterization of CNC batch #1–3, Figure S4: Effect of excess acid on measured conductivity, Figure S5 to Figure S8: DOE goodness of fit analysis. Table S1: DOE reaction conditions and results, Table S2: ANOVA for DOE response, Table S3: Final equation in terms of actual factors, and Equation S1: DOE generated equation in terms of coded factors.
