# Supplementary material for: Alkali Hydrolysis of Sulfated Cellulose Nanocrystals: Optimization of Reaction Conditions and Tailored Surface Charge
Source: Nanomaterials (Basel). 2019 Aug 30;9(9):1232. doi: 10.3390/nano9091232 (PMC6780348; doi:10.3390/nano9091232)
Supplement: Supplementary file 1 [file nanomaterials-09-01232-s001.pdf]

nanomaterials

Supporting Information for:

Alkali hydrolysis of sulfated cellulose nanocrystals: Optimization of reaction conditions and tailored surface charge

Jacobs H. Jordan[1], Michael W. Easson[1,*], and Brian D. Condon[1]

[1] The Southern Regional Research Center, Agricultural Research Service, USDA, 1100 Robert E. Lee Blvd., New Orleans, LA 70124, USA

* Correspondence: Michael.easson@usda.gov; Tel.: +01-504-286-4493

Table of Contents

List of Figures:

Figure S1. Characterization of CNC batch #3 used for alkali hydrolysis: (**a**) Representative 4 × 4 μm AFM height image of CNCs (0.01 wt%) on mica disc; (**b**) AFM height histogram; (**c**) AFM length histogram 2

Figure S2. Characterization of CNC batch #2 used for alkali hydrolysis: (**a**) AFM height histogram; (**b**) AFM length histogram 2

Figure S3. Characterization of CNC batch #1 used for alkali hydrolysis: (**a**) AFM height histogram; (**b**) AFM length histogram 2

Figure S4. Effect of excess acid on measured conductivity: Measured conductivity (corrected for Cl^-) from titration of 0.01 M HCl into 0.1 (black), 0.2 (red), 0.3 (blue), 0.4 (green), or 0.5 (purple) L cells. Solid lines are the theoretical values, based on the molar conductivity of the hydronium ion (H_3O^+, 349.6 $S{\cdot}cm^2{\cdot}mol^{-1}$) 3

Figure S5. Box-Cox analysis of DOE results for output sulfate remaining ($mmol{\cdot}kg^{-1}$). 6

Figure S6. Predicted *vs.* experimental $–OSO_3^-$ from DOE output, sulfate remaining ($mmol{\cdot}kg^{-1}$) 6

Figure S7. Residuals *vs.* predicted from DOE output, sulfate remaining ($mmol{\cdot}kg^{-1}$). 7

Figure S8. Normalized residuals plot showing the residuals are normally distributed 7

List of Tables:

Table S1. DOE reaction conditions and results. 4

Table S2. ANOVA for response, surface reduced quadratic model. 5

Table S3. Final equation in Terms of Actual Factors: 5

Figures, Tables, & Data:

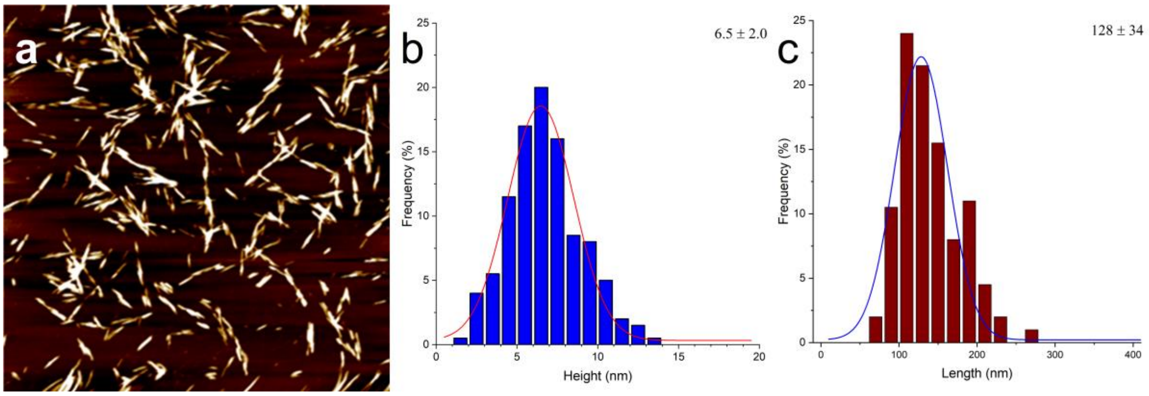

Figure S1. Characterization of CNC batch #3 used for alkali hydrolysis: (**a**) Representative 4 × 4 µm AFM height image of CNCs (0.01 wt%) on mica disc; (**b**) AFM height histogram; (**c**) AFM length histogram

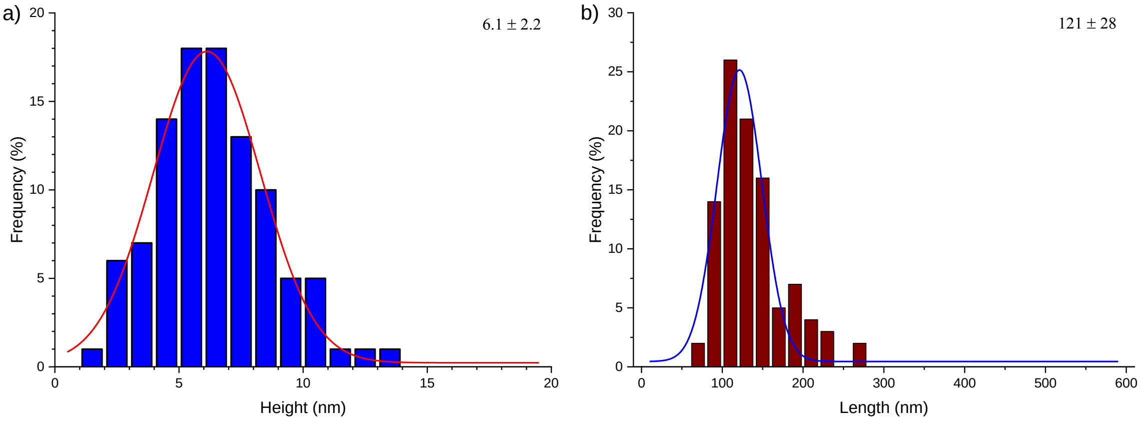

Figure S2. Characterization of CNC batch #2 used for alkali hydrolysis: (**a**) AFM height histogram; (**b**) AFM length histogram

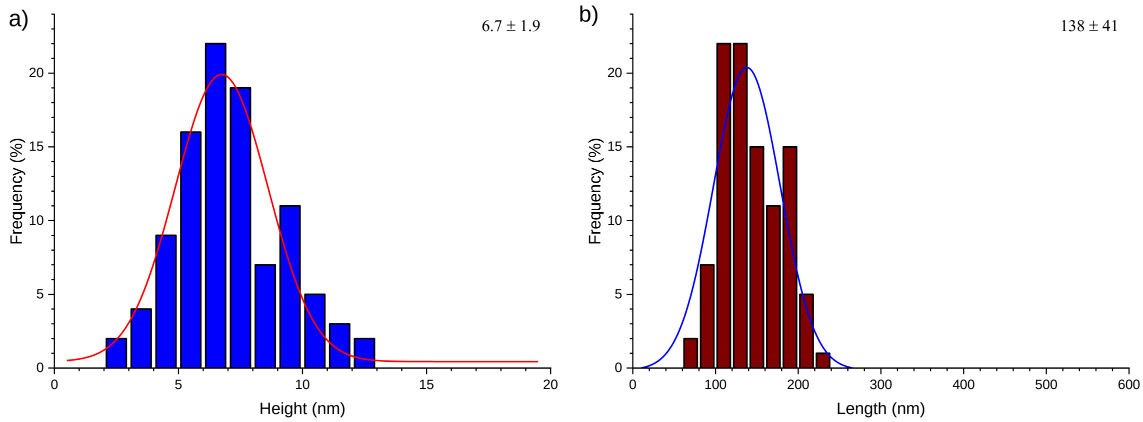

Figure S3. Characterization of CNC batch #1 used for alkali hydrolysis: (**a**) AFM height histogram; (**b**) AFM length histogram

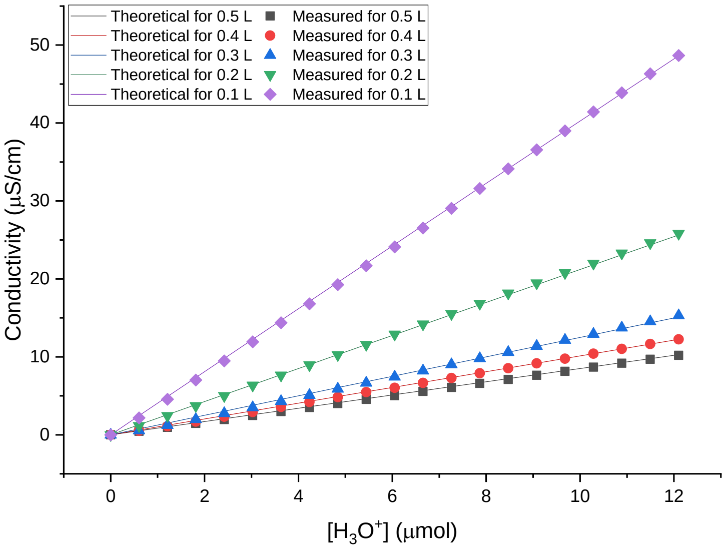

Figure S4. Effect of excess acid on measured conductivity: Measured conductivity (corrected for Cl^-) from titration of 0.01 M HCl into 0.1 (black), 0.2 (red), 0.3 (blue), 0.4 (green), or 0.5 (purple) L cells. Solid lines are the theoretical values, based on the molar conductivity of the hydronium ion (H_3O^+, 349.6 $S{\cdot}cm^2{\cdot}mol^{-1}$)

Table S1. DOE reaction conditions and results.

exp (#)	Time (min)	Temp (°C)	[NaOH] (M)	[CNC] (wt%)	Yield (%)	$-OSO_3^-$ (mmol·kg^{-1}) initial	final	Δ–OSO_3^- (%)
3	0	25	0.000	0.50	51%	211	204	3%
4	360	75	0.000	0.50	85%	197	193	2%
11	207	56	1.240	2.00	51%	211	92.8	56%
12	360	50	1.050	1.05	85%	211	61.6†	69%
13	207	56	1.240	2.00	62%	211	139.5	34%
14	0	75	2.000	0.50	97%	211	203.5	4%
15	360	25	2.000	2.00	73%	211	141.4	33%
16	74	53	0.100	0.50	80%	197	130.5	34%
17	167	25	0.800	2.00	67%	197	123.0	38%
18	0	25	0.100	1.51	68%	197	186.3	5%
19	0	75	0.100	2.00	32%	197	183.0	7%
20	360	25	0.100	0.50	65%	197	142.7	28%
21	360	75	2.000	2.00	48%	211	53.3	75%
22	0	46	2.000	2.00	62%	211	156.0	26%
23	166	25	2.000	1.06	53%	211	177.7	16%
24	0	25	1.390	0.50	59%	211	206.8	2%
25	54	50	1.390	1.13	77%	211	151.8	28%
26	0	75	0.610	1.06	68%	197	148.4	25%
27	360	75	0.100	0.50	64%	197	69.7	65%
28	74	53	0.100	0.50	68%	197	131.5	33%
29	360	50	1.050	1.25	78%	197	31.2	84%
30	360	54	0.100	2.00	95%	197	85.5	57%
31	54	50	1.390	1.13	79%	211	127.4	40%
32	7	75	1.380	1.88	71%	211	109.6†	48%
33	212	75	1.260	0.72	45%	211	81.5†	61%
34	360	54	2.000	0.50	76%	211	59.2	72%
35	166	25	2.000	1.06	81%	211	149.6	29%
36	360	50	1.050	0.50	63%	197	80.3†	62%
37	360	50	0.075	0.50	76%	197	109.1	45%
38	360	50	0.025	0.50	87%	197	170.3	14%
39	360	50	0.050	0.50	69%	197	155.6	21%
40	360	50	0.050	0.50	74%	197	147.9	25%
DOE TEST	360	60	0.130	0.78	77%	211	91.6	57%

The $\Delta(-OSO_3^-)$ standard error for (coefficient of variation, CV) in each instance is ~5% † indicates error was >10%; all other error was ≤10%. [1] The stock CNC suspensions were concentrated to ~3 wt% and diluted as necessary.

The Model F-value of 6.11 implies the model is significant. There is only a 0.02% chance that an F-value this large could occur due to noise. Values of "Prob > F" less than 0.0500 indicate significant model terms: A, B, AC, C^2. Values >0.10 indicate model terms are not significant (Table S2). The "Lack of Fit F-value" of 1.59 implies the Lack of Fit is not significant relative to the pure error (i.e. there is a 32% chance that the lack of fit occurred due to noise). The empirical model was tested with a confirmation run (e.g. DOE TEST) to confirm the results. Expected values were 83.2 mmol·kg^{-1} and 61%, and results gave a $\Delta(-OSO_3^-)$ of 57% within the standard error $\Delta(-OSO_3^-)$ of 5% and absolute error ($-OSO_3^-$ mmol·kg^{-1} ≤ 10%).

Table S2. ANOVA for response, surface reduced quadratic model.

Analysis of variance table [Partial sum of squares - Type III]						
Source	Sum of Squares	df	Mean Square	F Value	p-value Prob > F	
Model	53455.06	10	5345.51	6.11	0.0002	*significant*
A-Time	*28160.51*	*1*	*28160.51*	*32.20*	*< 0.0001*	*significant*
B-Temp	*8763.31*	*1*	*8763.31*	*10.02*	*0.0047*	*significant*
C-NaOH	*1148.74*	*1*	*1148.74*	*1.31*	*0.2647*	
D-CNC wt%	*792.54*	*1*	*792.54*	*0.91*	*0.3520*	
AC	*9146.78*	*1*	*9146.78*	*10.46*	*0.0040*	*significant*
AD	*1286.83*	*1*	*1286.83*	*1.47*	*0.2386*	
BC	*1713.29*	*1*	*1713.29*	*1.96*	*0.1762*	
CD	*1318.04*	*1*	*1318.04*	*1.51*	*0.2332*	
B^2	*1981.96*	*1*	*1981.96*	*2.27*	*0.1471*	
C^2	*6331.00*	*1*	*6331.00*	*7.24*	*0.0137*	*significant*
Residual	18368.35	21	874.68			
Lack of Fit	*15343.87*	*16*	*958.99*	*1.59*	*0.3209*	*not significant*
Pure Error	*3024.48*	*5*	*604.90*			
Cor Total	71823.41	31				

Results of the DOE generated the following equation (S1) in terms of coded factors:

$$107.77 - 49.07t - 35.41T + 39.37C + 24.13w - 33.13tC - 9tw - 21.85TC + 38.95Cw + 17.01T^2 + 67.35C^2 \quad \text{(S1)}$$

Where, time (t), Temperature (T), NaOH concentration (C), and CNC wt% (w) are the input factors.

Eq. S1 in turn is used to derive an equation in terms of actual factors (Table S3) based on input values.

Table S3. Final equation [(1)] in terms of actual factors:

sulfate remaining, $(-OSO_3^-)$ =	
+256.71715	
$-5.67961E{-}003 \times (t)$	Time
$-3.26401 \times (T)$	Temperature
$-0.077399 \times (C)$	NaOH Molarity
$-0.13912 \times (w)$	[(2)] sulfate half-ester (μmol)
$-2.00767E{-}004 \times T \times C$	
$-1.81883E{-}003 \times T \times w$	
$-8.73806E{-}004 \times T \times C$	
$+1.29817E{-}003 \times C \times w$	
$+0.027213 \times (T)^2$	
$+6.73481E{-}005 \times (C)^2$	

Notes: [1]The coefficients of each factor should not be used to determine their respective relative importance, since the units of each factor are not the same. [2] sulfate half-ester = starting sulfate (mmol·kg^{-1}) × CNC wt% × mass solution (kg) × 1000 μmol/mmol. e.g. 2.0 wt% × 200 mmol·kg^{-1} $(-OSO_3^-)$ × 0.020 kg H_2O × 1000 = 80 μmol.

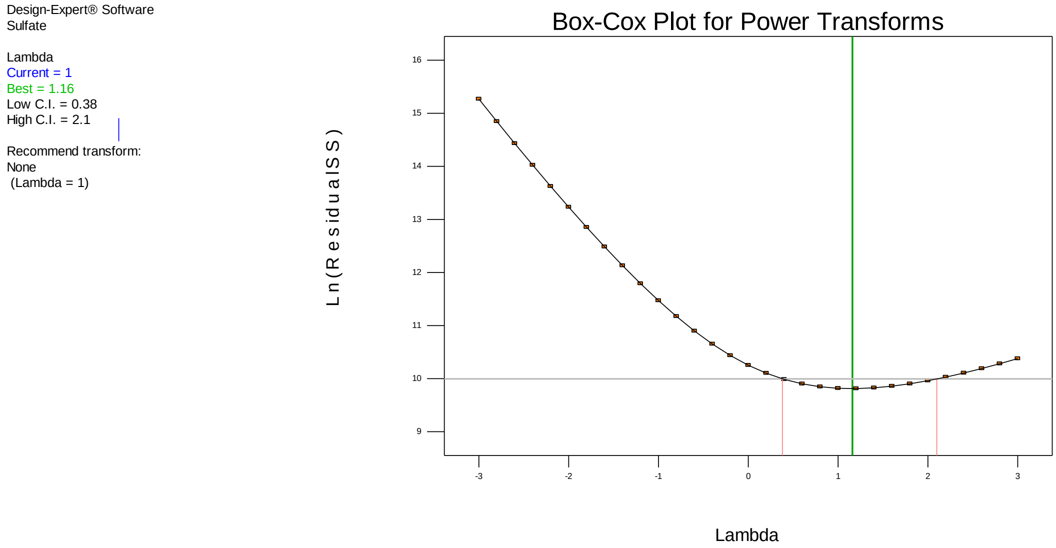

Figure S5. Box-Cox analysis of DOE results for output sulfate remaining (mmol·kg^{-1}).

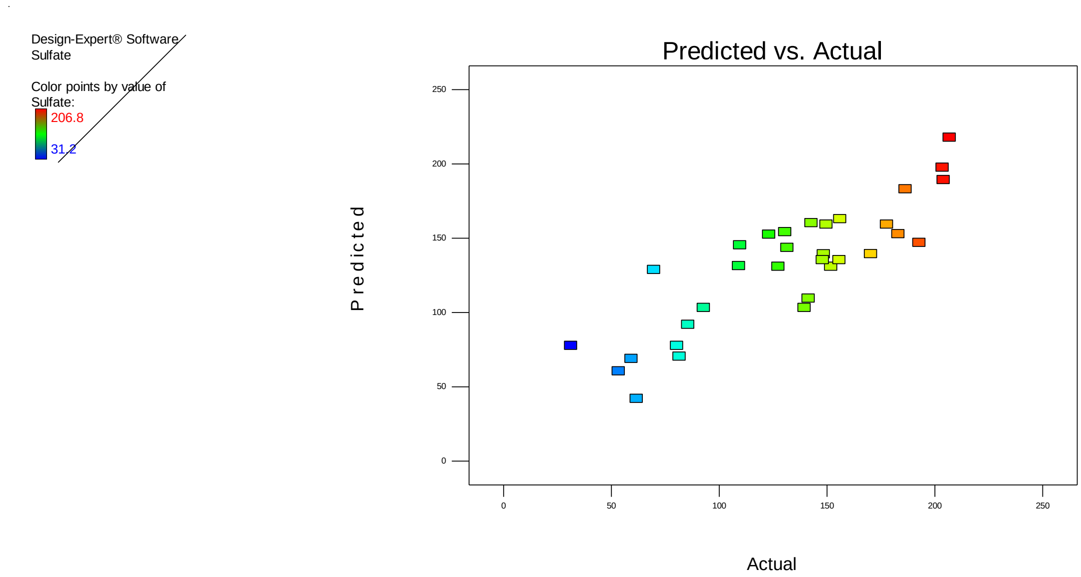

Figure S6. Predicted *vs.* experimental –OSO_3^- from DOE output, sulfate remaining (mmol·kg^{-1}).

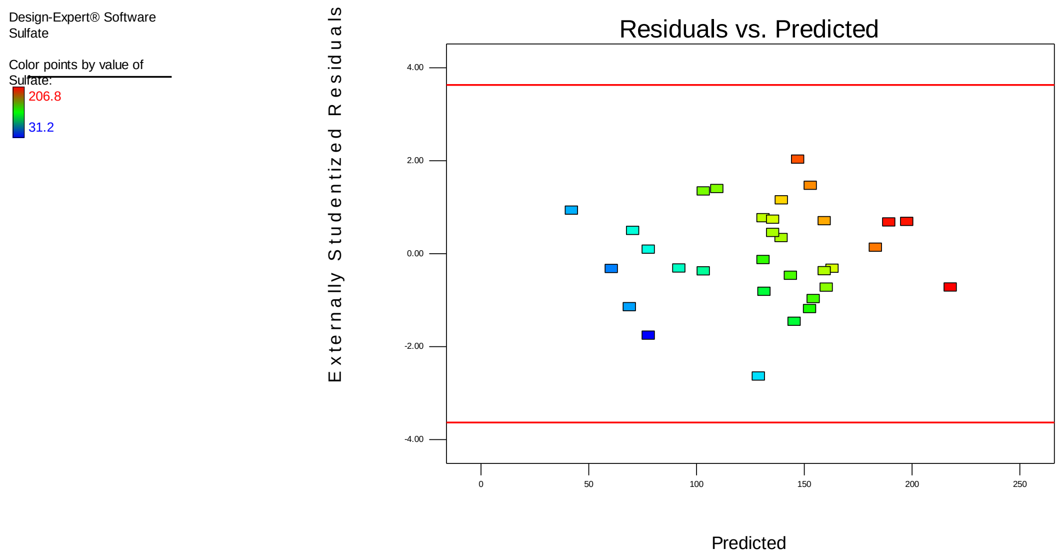

Figure S7. Residuals *vs.* predicted from DOE output, sulfate remaining (mmol·kg^{-1}).

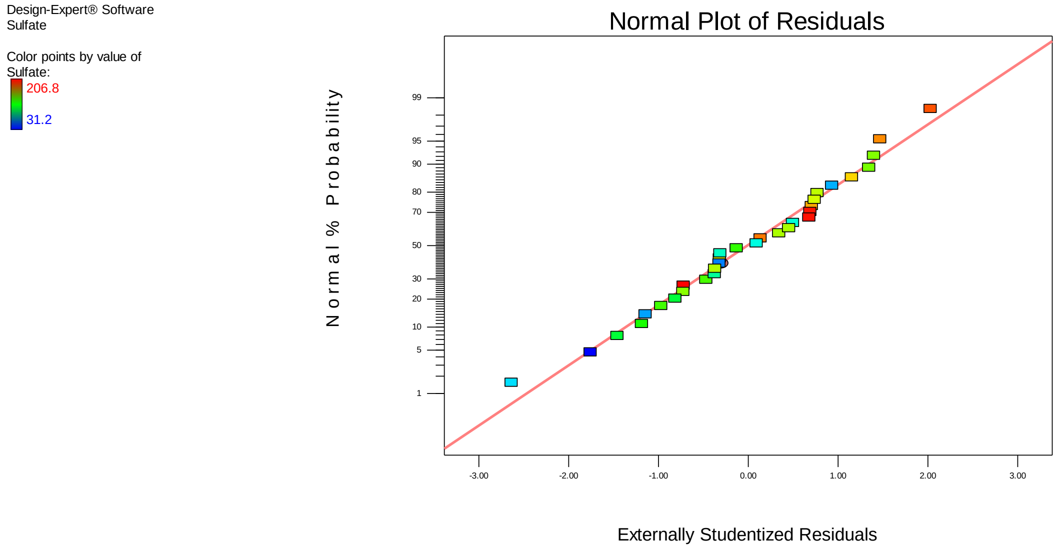

Figure S8. Normalized residuals plot showing the residuals are normally distributed

© 2019 by the authors. Submitted for possible open access publication under the terms and conditions of the Creative Commons Attribution (CC BY) license (http://creativecommons.org/licenses/by/4.0/).
